# The effect of mindfulness on social media addiction among Chinese college students: A serial mediation model

**DOI:** 10.3389/fpsyt.2023.1087909

**Published:** 2023-03-23

**Authors:** Hongming Chang, Xiaolu Meng, Yaqi Li, Jiaxi Liu, Wen Yuan, Jian Ni, Chunlu Li

**Affiliations:** ^1^Department of Psychology, School of Medical Humanitarians, Guizhou Medical University, Guiyang, China; ^2^Guizhou Health Development Research Center, Guiyang, China; ^3^Department of Applied Psychology, Hunan University of Chinese Medicine, Changsha, China

**Keywords:** mindfulness, social media addiction, attention control, fear of missing out, college students

## Abstract

**Introduction:**

The COVID-19 pandemic has exacerbated social media addiction (SMA), making it urgent to find effective interventions for social media addiction. Evidence has shown that mindfulness might be an effective intervention for social media addiction. However, psychological mechanisms by which mindfulness reduce social media use remain unclear. Here, we further addressed this issue to examine whether attentional control and fear of missing out (FOMO) mediate the relationship between mindfulness and SMA.

**Methods:**

We recruited 446 college students from two universities in China and analyzed the data.

**Results:**

The results suggest that there are mediation effects of attentional control and FOMO between mindfulness and SMA through 3 paths: path 1, mindfulness → attention control → SMA (−0.04); path 2, mindfulness → FOMO → SMA (−0.22); and path 3, mindfulness → attention control → FOMO → SMA (−0.05).

**Discussion:**

Therefore, mindfulness-based interventions may be an effective way to alleviate social media addiction, especially mindfulness-based interventions targeting FOMO. At the end of the article, we also discussed the limitations of this study.

## Introduction

Social media use has increased exponentially. The number of social media users is estimated to have reached 3 billion worldwide (1). Social media facilitates social interaction and lowers stress for at least some users, but overuse can lead to serious negative consequences, such as social media addiction (SMA), poor sleep quality, less offline social interaction, and more depression and anxiety symptoms (2–5). The estimated SMA prevalence of college students is around 23% (6). To make matters worse, the COVID-19 outbreak exacerbated social media addiction and its negative consequences, such as sleep problems (7, 8), the negative impact on the well-being of excessive social media users (9), increased daily stress, and an increase in suicide-related outcomes (10, 11). Given its high prevalence and severe negative consequences, the question of how to alleviate social media addiction and reduce the adverse consequences of social media addiction has become very urgent.

Many antecedents leading to social media addiction have been investigated, such as attachment styles (12), self-esteem (13, 14), the need to socialize (15), and social anxiety (13). However, how social media addiction can be systematically controlled or regulated remained unsolved in these studies.

Existing evidence suggests that mindfulness-based interventions may be an effective intervention for social media addiction. Mindfulness helps us to be aware of where we are and what we are doing rather than to be overreacting or being overwhelmed by what is happening around us (16). It is a basic human ability to be fully present and prevalent even among ordinary people who have not been systematically trained in mindfulness (17). The ability to sustain mindfulness is called trait mindfulness (TM) (18), which can be improved by mindfulness training (19). Research interested in mindfulness-based interventions (MBI) has exploded over the past few decades (20). Mindfulness has been shown to be negatively associated with social media addiction (21–24), and has an indirect effect on social media addiction through mediation (25, 26) or moderation (27–29). However, the psychological mechanisms by which mindfulness reduces social media addiction remain unclear. These psychological mechanisms can help us design mindfulness-based interventions more effectively.

Two studies have shown that the influence of mindfulness to reduce social media addiction is mediated by the ability of social pressure self-efficacy (29), positive effects on self-esteem, and negative effects on social anxiety (13). In the present study, based on core features of mindfulness and social media addiction, we focused on the potential role of attentional control and social media-related fear of missing out (FOMO).

### The mediating effect of fear of missing out

Currently, the COVID-19 pandemic remains a global challenge. Restrictions on the movement of people are one of the government policies aimed at reducing the spread of COVID-19. In this context, the pandemic dramatically changed one’s usual routine and decreased social contacts with each other, ultimately leading to fearful experiences that are particularly relevant to interpersonal interactions, especially the fear of missing out (FOMO). FOMO is defined as a widespread anxiety arising when individuals cannot get the experiences they want to know about, mainly manifested by a persistent desire to know what others are doing (30). In fact, it has been shown that the COVID-19 pandemic increased the fear of FOMO in China (31). During lockdowns, social media use may be one of the most effective and available ways to ease FOMO. The latter has been highlighted in previous literature as an important influencing factor of social media use (32, 33), including problematic social media use (34, 35). Therefore, FOMO may be a significant contributor to social media addiction, especially under the COVID-19 pandemic.

FOMO is likely to distract people from their experiences of in-the-moment. In fact, when one is worrying about what one is missing out, it will become especially difficult to focus on what one is doing now. Conversely, when a person is deeply involved in what they are doing at the moment, they may be less likely to think of alternatives and then will feel less FOMO (36). Mindfulness practice places a strong emphasis on focusing on the here and now. This suggests that the fear of missing out is an important target for mindfulness interventions. In fact, related research has provided direct evidence for the relationship between mindfulness and fear of missing out. For example, less mindful attention was associated with higher levels of FOMO (37). Mindfulness practice has reduced levels of fear of missing out (38). Based on this evidence, we hypothesized that FOMO mediates the effect of mindfulness on social media addiction (H1). To be specific, mindfulness may decrease FOMO of Chinese college students, which, in turn, may decrease social media addiction.

### The serial mediating role of attention control and FOMO

The psychological mechanisms by which mindfulness reduces fear of missing out in the context of social media addiction require further exploration. Theoretically, attention is widely regarded as a core aspect of mindfulness (39, 40). And the practice of self-regulation of attention is one common component among various types of mindfulness-based interventions (41). For example, mindfulness meditation typically involves focusing attention on a selected target object and redirecting attention on the same target object in the event of distraction (39). In fact, attentional enhancement is one of the most common outcomes of different types of mindfulness-based interventions (40). For example, 8 weeks of mindfulness practice on novice college students has been shown to improve their alertness (39), attentional orienting (39, 42), and attention executive control (42).

According to the process model of emotion regulation, individuals regulate their emotion in the situation-attention-appraisal-response sequence, and attentional deployment is a cardinal emotion regulation strategy (43, 44). Human attention is usually deployed voluntarily based on observers’ goals (top-down) or shifted automatically according to noticeable features of stimuli in the environment (bottom-up). The former is usually called attentional control and is regulated by the anterior system, while the latter is usually called attention orientation, such as attention bias, which is regulated by the posterior system (45). However, a recent study found that there is no attentional bias toward relevant information in social media addiction (46). These evidences suggested that attentional control might be central to FOMO regulation.

Besides, a number of studies have found that reduced attention control ability affected individuals’ social media use. For example, attention deficit among college students significantly and positively predicted the severity of social media addiction (47, 48). Cognitive flexibility and sustained attention have been significantly and negatively associated with social media addiction in college women (49). Moreover, attention control deficits have become a risk marker for the anxiety onset and persistence (50, 51). Impaired attention control makes individuals more likely to skew attentional resources toward threatening information in the presence of anxiety (52). As an anxious emotion, the negative association between FOMO and attention control has been verified by previous studies (26, 38, 53).

Furthermore, according to the Interaction of Person-Effect-Cognition-Execution (I-PACE) model proposed by Brand (54), when executive functions (e.g., attention control) are weak, the conditioned learning effects of negative emotions, such as anxiety, can cause social media use to gradually get out of control and deteriorate into compulsive use (55, 56). Thus, we hypothesized that mindfulness may indirectly influence social media addiction through the paths of attention control (H2) and attention control → FOMO (H3).

### The present study

**Figure 1:**
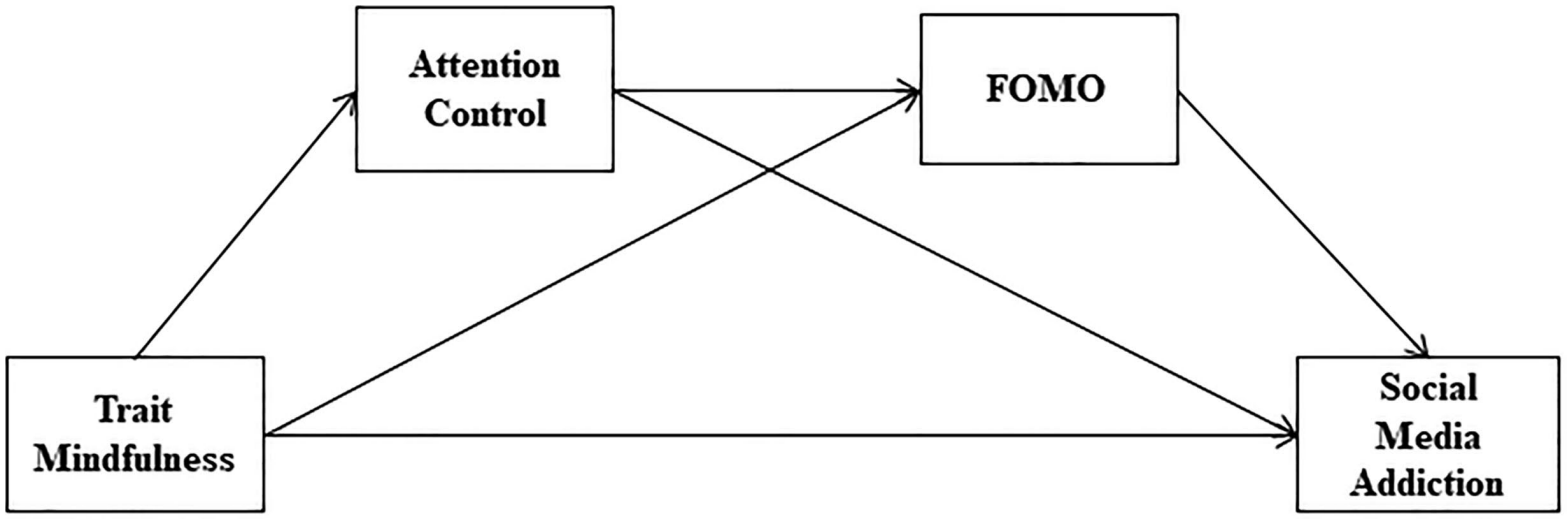
The serial mediation model hypothesized in the present study.

Based on the aforementioned literatures, the present study aimed to explore the psychological mechanisms underlying the effect of mindfulness on social media addiction. First, we recruited Chinese college students and examined the correlation between their mindfulness and social media addiction. Then, we further tested whether attention control and FOMO independently mediate this association. At the end, we investigated whether attentional control and FOMO mediate the relationship between mindfulness and social media addiction in a serial way. In conclusion, we aimed to fill in the gaps in the relationship between these variables, with the hope of providing a theoretical basis for intervention research in this context. We showed the hypothetical model in Figure 1.

## Materials and methods

### Participants

We conducted online and offline questionnaire surveys at two universities in China: Guizhou and Hunan, respectively. A total of 502 students were enrolled by stratified random sampling. All participants voluntarily filled out the questionnaire to participate in the study, without receiving rewards. All the participants were asked to sign informed consent forms and were told that they were participating in the study voluntarily and could terminate the questionnaire freely throughout the process. To ensure data quality, we excluded the data from two subject groups: (1) those who chose the same answer for greater than or equal to half the length of the total scale (57) and (2) those who did not sign the informed consent form. Finally, data from 446 participants (120 male and 326 female) ranging in age from 18 to 26 years (M = 20.36, SD = 1.40) were analyzed. This research was approved by the Ethics Committee of Guizhou Medical University.

Demographics are shown in Table 1. 26.9% and 73.1% of the participants were males and females, respectively. The ages of the participants ranged from 17 to 26 years (mean = 20.36, SD = 1.40). 22% of the participants came from cities, 27.6% came from towns, and 50.4% came from villages. Among all the participants, “only-child” (i.e., participants who were an only-child) accounted for 18.2%, while “non-only child” accounted for 81.8%; 44.4% were “left-behind child” (i.e., participants who experienced both or one parent relocating elsewhere to work for more than 6 months while the participant was growing up), while the rest, 55.6%, were not “left-behind child.”

**Table 1:** Descriptive statistics.

Variable		*N*	(%)	Variable		*N*	(%)
Gender	Male	326	73.09	Left-behind child	Yes	198	44.39
	Female	120	26.91		No	248	55.61
Residence	City	98	21.97	Only-child	Yes	81	18.16
	Town	123	27.60		No	365	81.84
	Village	225	50.44				

## Measures

### Social media addiction

To measure the social media addiction of participants, the Bergen Facebook Addiction Scale (BFAS) was used (58), which was translated into the Chinese version by Yubo Hou (59). These items relate to experiences that have occurred in the past year and are rated on a 5-point scale from 1 (Very rarely) to 5 (Very often; example item: “ignored your partner, family members, or friends because of social media?”). The scale consists of 6 dimensions: salience, tolerance, mood changes, relapse, withdrawal, and conflict. In view of the characteristics of social networking sites in mainland China, we replaced the original scale, namely Facebook, Twitter, Instagram, etc., with those social networking sites that are popular in China: QQ, Weibo, WeChat, etc., in the instructions. The scores of the participant for all the items were added to form a social media addiction score, with higher scores indicating higher levels of social media addiction. Cronbach’s α of the whole scale was 0.92 in the current sample.

### Mindfulness

To measure mindfulness, the Chinese version of the Mindful Attention Awareness Scale (60, 61) was used. The scale consists of 15 self-rating items on everyday experiences. An example is the item “I tend not to notice feelings grab my attention.” Responds rated each item on a 6-point Likert-type scale, with a range from 1 (almost always) to 6 (almost never). Total scores can range from 15 to 90, with higher scores indicating a greater level of mindfulness. In this study, Cronbach’s alpha was 0.86.

### Attention control

To measure attention control, we used the Attention Control Scale (62), which was translated into the Chinese version by Siying He (63). The questionnaire has two dimensions: focusing (9 items) and shifting (11 items). Each item of this scale was rated on a 4-point scale from 1(almost never) to 4 (almost ever; i.e., almost always). In the present study, the Cronbach’s alpha of the Attention control Scale was 0.76.

### The fear of missing out

To measure FOMO, we used the Fear of Missing Out in the Mobile Social Media Environment Measurement Scale, compiled by Song Xiaokang (64). The scale measures individual FOMO in the context of social media use, and consists of four dimensions: psychological motivation (e.g., “On mobile social media, as soon as I see a hint of what’s new, I’m eager to click on it right away.”), cognitive motivation (e.g., “Using mobile social media, I was able to get the news, business, or expertise I wanted”), behavioral performance (e.g., “As soon as I have time, such as waiting for the bus or recess, I am used to turning on mobile social media to check for new news or updates”), and emotional dependence (e.g., “If I cannot use mobile social media for a few days, I feel lost”). This scale has 16 items. Each item was rated on a 5-point Likert scale (1 = not at all true to 5 = absolutely true). The total score was between 16 and 80, and the higher the total score, the greater the fear of missing out. The Cronbach’s alpha was 0.87, in this study.

### Statistical analysis

In the present study, data were analyzed according to how Hayes (65) described the process in his book on the SPSS 26.0. First, descriptive statistics of the investigated variables were calculated. Then, to examine the bivariate correlations between mindfulness, social media addiction, FOMO, and attention control, Pearson’s correlation analysis was used. Additionally, we ran PROCESS Macro Model 6 to examine simultaneously all the study hypotheses, as presented in Figure 1. To determine the magnitude of these effects, the bootstrapping method based on 5,000 re-samples of the data was used to produce 95% bias corrected confidence intervals (Cis). When Cis do not overlap with zero, the effect is significant at α = 0.05.

## Results

### Common method biases tests

To exclude common method bias, (66) single-factor test and an exploratory factor analysis were performed, where all items including four variables were included. We found that the first factor accounted for 20.7% of the total variance, which was below the 40% threshold proposed by Podsakoff et al. (67). Therefore, common method biases are unlikely to confuse the interpretation of the data analysis.

### Descriptive analysis and correlations between overall variables

Table 2 presents basic descriptive data on mindfulness, attention control, FOMO, and social media addiction. The mean total scores of mindfulness were 57.43 ± 10.62 (range = 25 to 87), the meant total scores for attention control were 49.87 ± 6.05 (range = 28 to 72), the mean total scores for FOMO were 54.8 ± 9.44 (range = 27 to 78), and the mean total scores for social media addiction were 46.35 ± 12.51 (range = 20 to 83).

**Table 2:** Descriptive statistics and results of correlational analysis of variables (*N* = 446).

	*M*	*SD*	1	2	3	4
1. Mindfulness	57.43	10.62	1			
2. Attention control	49.87	6.05	0.40^**^	1		
3. FOMO	54.80	9.44	−0.44^**^	−0.35^**^	1	
4. Social media addiction	46.35	12.51	−0.52^**^	−0.37^**^	0.67^**^	1

**p < 0.01.

To test the bivariate correlations of all the variables, we conducted a Pearson’s correlation analysis where standard scores for each variable were used. As shown in Table 2, all the variables were significantly correlated with each other, *p* < 0.01. Mindfulness was significantly and positively correlated with attention control (*r* = 0.40, *p* < 0.01), while significantly negatively correlated with FOMO and SMA (*r* = −0.44, *p* < 0.01; *r* = −0.52, *p* < 0.01), respectively. Attention control was significantly negatively correlated with FOMO and SMA (*r* = −0.37, *p* < 0.01; *r* = −0.37, *p* < 0.01), respectively. FOMO was significantly positively correlated with SMA (*r* = 0.67, *p* < 0.01).

### Testing the serial mediating effects

**Figure 2:**
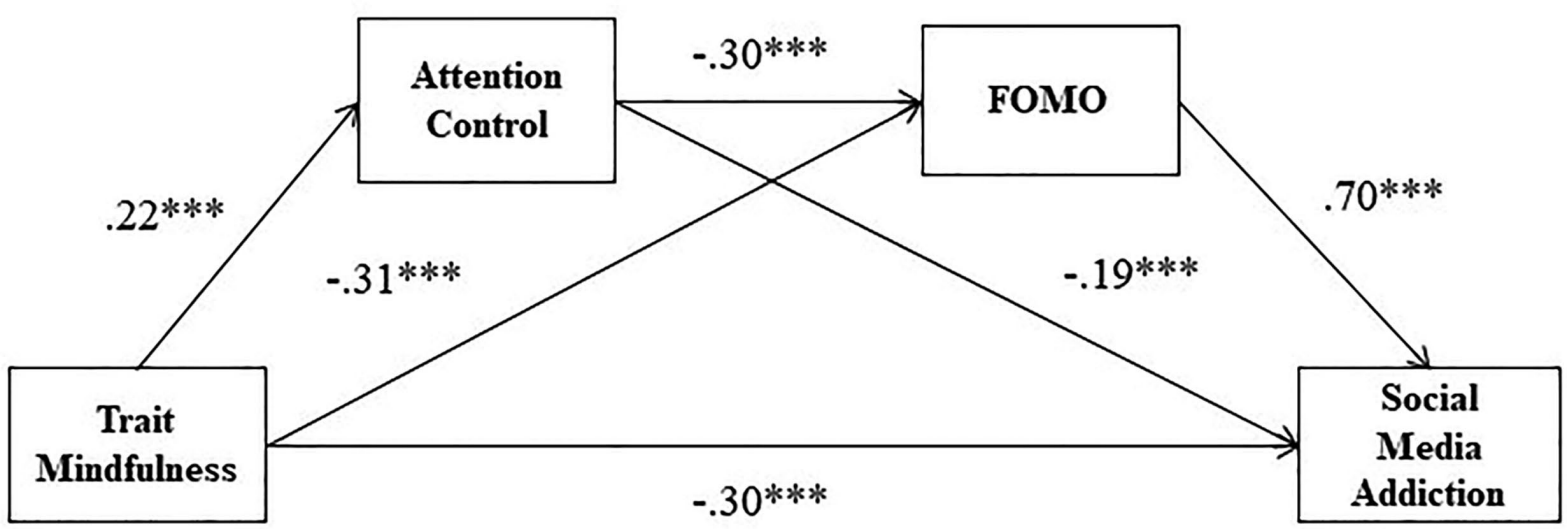
Theoretical research model with standard coefficients. Regression coefficients were obtained with gender, age, place of residence, only-child, and left-behind child, in covariates in PROCESS Procedure for SPSS, where *** denotes that *p* < 0.001, *N* = 446.

Mindfulness, attentional control, FOMO, and social media addiction are all significantly associated, meeting the statistical requirements for a further mediation analysis of mindfulness and SMA (68). With gender, age, place of residence, only-child, and left-behind child as covariates, the mediating role of attention control and FOMO in the relationship between mindfulness and SMA was analyzed by using PROCESS Model 6 in SPSS 26.0 compiled by Hayes (65). The main results are shown in Tables 3, 4. As is shown in Table 3, Mindfulness was a significant predictor of attention control (*β* = 0.22, *SE* = 0.03, *p* < 0.001). Mindfulness and attention control are also significant predictors of FOMO (*β* = −0.31, *SE* = 0.04, *p* < 0.001; *β* = −0.31, *SE* = 0.07, *p* < 0.001). Finally, trait mindfulness and attention control, as well as FOMO, significantly predicted social media addiction (*β* = −0.30, *SE* = 0.05, *p* < 0.001; *β* = −0.19, *SE* = 0.08, *p* = 0.01; *β* = 0.70, *SE* = 0.05, *p* < 0.001). The results of the path coefficient test in the hypothetical model are shown in Figure 2.

**Table 3:** Regression coefficients, standard errors, and model summary information for the influence of mindfulness in a model of social media addiction.

Antecedent	Consequent										
	Model 1 (Attention control)				Model 2 (FOMO)				Model 3 (Social media addiction)		
	Coefficient	*SE*	*p*		Coefficient	*SE*	*p*		Coefficient	*SE*	*p*
Gender	0.98	0.60	0.11		−2.09	0.89	0.02		1.25	0.96	0.19
Age	0.13	0.20	0.51		−0.06	0.30	0.83		−0.16	0.32	0.61
Left-behind child	0.42	0.55	0.44		−1.98	0.81	0.02		−1.31	0.87	0.13
Residence	−0.31	0.34	0.37		−1.38	0.51	<0.001		−0.30	0.54	0.58
Mindfulness	0.22	0.03	<0.001		−0.31	0.04	<0.001		−0.30	0.05	<0.001
Attention control	-	-	-		−0.30	0.07	<0.001		−0.19	0.08	0.01
FOMO	-	-	-		-	-	-		0.70	0.05	<0.001
Constant	34.32	4.23	<0.001		95.83	6.68	<0.001		40.20	8.65	<0.001
	*R*^2^ = 0.17				*R*^2^ = 0.26				*R*^2^ = 0.52		
	*F* (4, 441) = 22.07, *p* < 0.001				*F* (5, 440) = 29.58, *p* < 0.001				*F* (6, 439) = 79.08, *p* < 0.001		

**Table 4:** Direct and indirect effects of mindfulness on social media addiction.

Path way	Estimate	*SE*	*p*	95% CI	Relative effect(%)
Total effect	−0.61	0.05	<0.001	−0.70, −0.51	-
Direct effect	−0.30	0.05	<0.001	−0.38, −0.21	48.60
Total indirect effect	−0.31	0.04	<0.001	−0.38, −0.24	51.40
Mindfulness → FOMO → Social media addiction	−0.22	0.03	<0.001	−0.29, −0.16	36.68
Mindfulness → attention control → Social media addiction	−0.04	0.02	<0.001	−0.08, −0.01	6.88
Mindfulness → attention control → FOMO → Social media addiction	−0.05	0.01	<0.001	−0.08, −0.02	7.85

The mediating effect sizes of attention control and FOMO in the relationship between mindfulness and SMA are shown in Table 4. As shown in Table 4; Figure 2, the mediating role between the mindfulness and SMA of attention control and FOMO is significant. The total effect value of mindfulness on SMA was −0.61, and the direct effect value of mindfulness on SMA was −0.29, and the total mediating effect accounted for 51.40% of the total effect. The mediating effect consists of three indirect effects: Path 1: Mindfulness → FOMO → SMA (−0.22), Path 2: Mindfulness → Attention Control → SMA (0.04), and Path 3: Mindfulness → Attention Control → FOMO → Social Media Addiction (−0.05). For pathways 1, 2, and 3, the ratios of the three indirect effects to the total effect were 36.68%, 6.88%, and 7.85%, respectively. All three indirect effects reached a significance level, since CIs of the above indirect effects did not include the zero value. Thus, hypotheses 1, 2, and 3 were confirmed.

The above results suggest that mindfulness not only indirectly predicts SMA through the single mediating effects of attentional control and FOMO, but also indirectly predicts SMA through the serial mediating effects of attentional control and FOMO.

## Discussion

Mindfulness might be a protective factor, for college students, from social media addiction under the COVID-19 pandemic. However, psychological mechanisms by which mindfulness may reduce social media use remain unclear. The present study analyzed the relationship between mindfulness and social media addiction among Chinese college students *via* a serial mediation model. The results of this study suggest that attention control and fear of missing out partially mediate the relationship between mindfulness and social media addiction. Consequently, in this study, we confirmed a direct link between mindfulness and social media addiction in Chinese college students (38). We found that mindfulness, through three paths, influence social media addiction, and that these three paths are: attention control, fear of missing out, and attention control → fear of missing out. These results help us further our understanding of the relationship between mindfulness and social media addiction, and provide theoretical support for future mindfulness interventions for social media addiction.

Firstly, we demonstrated the mediating role of fear of missing out in mindfulness and social media addiction. Specifically, college students with higher levels of mindfulness had lower FOMO in the mobile social media environment, further reducing their possible SMA. According to the mechanisms of mindfulness (40), college students with higher levels of mindfulness might reduce SMA by allowing FOMO to settle, by identifying and accepting it. SMA and FOMO were found to be negatively related to mindfulness in the southeastern United States (26), India (69), and the Middle East (70) in previous researches. Consistently, similar relationships among SMA, FOMO, and mindfulness were also found in Chinese sample in our present study. Moreover, the effect of mindfulness on social media addiction through FOMO was the highest in this study, up to 36.68%. This percentage underscores the role of FOMO in how mindfulness affects the SMA process. This result suggests that reducing FOMO might be an important mechanism by which mindfulness changes social media addiction. However, no effect of a mindfulness intervention on the FOMO in the Middle East sample was found (70). This may be attributed to the effectiveness of mindfulness intervention program design and implementation. Because they did not design mindfulness interventions specifically for FOMO, and implemented them in the form of psychoeducation rather than group psychotherapy. Social Media Mindfulness Practice had been proposed recently (38).

On the other hand, the correlation between FOMO and SMA in this study is greater than that in previous studies (37, 71). This discrepancy may stem from the COVID-19 pandemic, which indicates that the relationship between FOMO and social media addiction might have become stronger under the COVID-19 pandemic. Whether this pathway is dependent on the COVID-19 scenario deserves further exploration in the future.

Secondly, this study found a significant pathway of mindfulness → attention control → fear of missing out on social media addiction. This model suggests that the serial relationship between attention control and fear of missing out mediates the relationship between mindfulness and social media addiction. Studies have shown that increased mindfulness improves attentional function (39, 72). In our results, the negative correlation between attention control and FOMO is similar to previous results (50, 73). That is to say, in general, the higher the mindfulness of college students, the higher their attention control function might be, which might potentially lead to them producing less FOMO, and their reduced FOMO may further reduce their potential social media addiction. Some evidence from neuroscience suggests that selective attention and the activation of its inhibitory attention-related networks are associated with the suppression of emotions (74). We hypothesize that this may be a continuum: i.e., higher levels of mindfulness may improve individuals’ attentional functions such as vigilance, sustained attention, and conflict monitoring, which may help identify some emotional precursors closely related to addictive behaviors and thus reduce the occurrence of addictive behaviors.

In terms of effect size, FOMO alone as a mediating role had the largest effect value, while attention control and FOMO as serial mediating roles has a small effect value. Therefore, the pathway of mindfulness → attention control → fear of missing out on social media addiction appears to play a limited role in mindfulness-based amelioration of social media addiction. On the other hand, these data suggest that mindfulness may reduce fear of missing out through other pathway, such as the non-judgment facet of mindfulness which was strongly and inversely related to negative affect and anxiety (75). Further research is needed in the future.

Finally, we verified the mediating effect of attention control on mindfulness and social media addiction. The results suggest that if college students have higher levels of mindfulness, their ability to control attention may also be higher. Such college students might effectively be able to control their attention, stay away from threatening information related to social media, and consequently reduce possible social media addiction (26). Previous studies have indicated that everyone can be distracted by excessive social media information, which can easily lead to attention fixation on social media information (76). However, a high level of mindfulness enables an individual to spot situations in which attention cannot be taken away in time (16). It seems that college students could also actively improve attention control ability to reduce the risk and behavior of social media addiction through Social Media Mindfulness practice. Because attention to current experiences and states is a core concept of mindfulness and can significantly enhance focused attention, which is one of the components of attention (77). However, the mediating effect size for attention control was small, and such results suggest that there might be more powerful mediating pathways for the effect of mindfulness on social media addiction. Therefore, when we design mindfulness for social media, we should not focus too much on attentional control.

This research has made several theoretical and practical contributions. On the one hand, we revealed that attention control and FOMO mediated the relationship between mindfulness and SMA by three paths, with an emphasis on the mediation of FOMO. On the other hand, our research suggests that mindfulness-based interventions might be an effective intervention to alleviate social media addiction, especially mindfulness-based interventions targeting FOMO.

## Limitations

Our study may have some limitations as follows. The first limitation is related to our sample. We only carried out the survey in Guizhou and Hunan provinces in southern China, and therefore, follow-up research should expand the scope of the survey and utilize more representative population data. The proportion of women in this study reached 73.1%. Although we included gender as a covariate to control, future research should avoid imbalances in the gender ratio. Secondly, the present study is a cross-sectional study, only reflecting the degree of correlation between studies. In the future, longitudinal comparison will explain the causal relationship between variables. Thirdly, we only examined the mediating role of attentional control, and other facets of mindfulness will need to be further explored in the future, such as non-judgment, non-reactivity, and acting with awareness. Finally, we did not do any mindfulness intervention, although the conclusions have practical implications, they need to be confirmed by future intervention studies.

## Conclusion

Mindfulness was significantly and negatively associated with social media addiction. Attention control and FOMO played mediating roles on the effect of mindfulness on social media addiction in parallel, with a large indirect effect value of FOMO, and a small indirect effect value of attention control. Attention control and FOMO also played mediating roles on the effect of mindfulness on social media addiction serially, but the indirect effect of serial mediating is small. Therefore, special emphasis should be placed on FOMO when designing social media mindfulness interventions.

## Data availability statement

The raw data supporting the conclusions of this article will be made available by the authors, without undue reservation.

## Ethics statement

The studies involving human participants were reviewed and approved by the Ethics Committee of Guizhou Medical University. The patients/participants provided their written informed consent to participate in this study.

## Author contributions

CL and HC designed the study. HC, XM, YL, JL, WY, and JN collected and analyzed the data. CL and HC wrote the manuscript. All authors contributed to the article and approved the submitted version.

## Funding

This research was funded by the Guizhou Medical University start-up fund for doctoral talent (J-[2021]050; J-[2021]051) and the National Natural Science Foundation of China (32260199).

## Conflict of interest

The authors declare that the research was conducted in the absence of any commercial or financial relationships that could be construed as a potential conflict of interest.

## Publisher’s note

All claims expressed in this article are solely those of the authors and do not necessarily represent those of their affiliated organizations, or those of the publisher, the editors and the reviewers. Any product that may be evaluated in this article, or claim that may be made by its manufacturer, is not guaranteed or endorsed by the publisher.
